# A Rare Case of Spinal Hypertrophic Pachymeningitis That Caused Tetraplegia After Myelography

**DOI:** 10.7759/cureus.75684

**Published:** 2024-12-13

**Authors:** Yoshiki Shimokawa, Shuhei Murase, Masataka Mitsuhashi, Motoo Shimizu, Kiyofumi Yamakawa

**Affiliations:** 1 Department of Orthopaedics, Tokyo Metropolitan Bokutoh Hospital, Tokyo, JPN; 2 Department of Orthopaedics, Nishikasai Minamiguchi Orthopaedic Clinic, Tokyo, JPN

**Keywords:** ana cytoplasmic antibody-associated vasculitis, eosinophilic granulomatosis with polyangiitis (egpa), hypertrophic pachymeningitis, myelography, tetraplegia

## Abstract

Hypertrophic pachymeningitis (HP) is a rare inflammatory disease that causes the thickening of the dura mater. Its etiology is mainly classified as idiopathic or secondary, and autoimmune disease is one of the main causes of secondary HP. Antineutrophil cytoplasmic antibody (ANCA)-associated vasculitis and IgG4-related disease are common among autoimmune diseases.

Here we present a case of spinal HP in which the patient showed spinal shock and neurological symptoms deteriorated after myelography. Since the patient was sedated without caution of the neck posture, the HP itself compressed the spinal cord, which led to the tetraplegia.

To our knowledge, this is the first case report of such an entity. Our case highlights the risk of sedation for patients who have hypertrophic lesions of the dura mater.

## Introduction

Hypertrophic pachymeningitis (HP) is a rare inflammatory disease that affects the central nervous system (CNS), cerebrum, and spinal dura mater. Hypertrophic pachymeningitis of the spine (HSP) is relatively rare compared to the cerebrum form [1]. Hypertrophic pachymeningitis is often classified as idiopathic and secondary, depending on whether there are any background etiologies or not. Antineutrophil cytoplasmic antibody (ANCA)-associated vasculitis is one of the various causes of secondary HP, as a local manifestation of the CNS [2]. Eosinophilic granulomatosis with polyangiitis (EGPA) is one of the ANCA-associated vasculitis, but HP as a local manifestation of the disease is even more uncommon [3, 4].

Spinal HP causes thickening of the dura mater with chronic inflammation and fibrillation pathologically, which causes various neurological symptoms such as neck or back pain, radiculopathy, and, in some cases, myelopathy [3]. Here we present a case of HP of the cervical spine that followed an atypical clinical course.

## Case presentation

An 80-year-old male patient presented to our Department of Neurology with an eight-month history of bilateral numbness in his lower extremities. The patient had suffered from asthma previously and showed purpura on admission. Sensory disturbance and muscle weakness at the distal muscle, which suggest the symptoms of peripheral neuropathy, occurred thereafter. Laboratory data revealed eosinophilia, which led to the diagnosis of EGPA. The treatment was initiated with 12.5 mg/day of prednisone.

**Figure 1:**
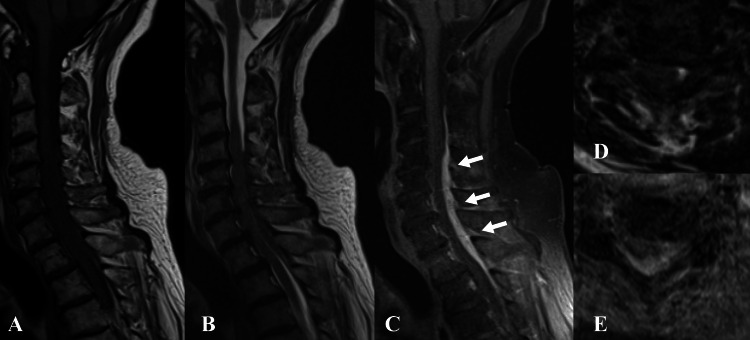
Preoperative gadolinium-enhanced MRI of the cervical spine The spinal cord is severely compressed by a mass lesion dorsally at the level of C4-Th1 (white arrows). The patient was unable to maintain the posture due to the neck pain, which resulted in blurry images. Aː Sagittal T1-weighted image; Bː Sagittal T2-weighted image; Cː Sagittal T1 fat-saturated weighted image; D: Axial plain T2-weighted image at the C4/5 level; E: Axial gadolinium-enhanced image at the C4/5 level

Two months after the treatment, headache, and gait disturbance occurred, and the patient was readmitted to the neurology department. Gadolinium-enhanced magnetic resonance imaging (MRI) of the cervical spine was performed, and it revealed an extradural mass lesion, compressing the spinal cord severely at the level of C4 to Th1 (Figure 1). The mass lesion, located dorsally, seemed to be a thickening of the dura mater. 

Considering the present history of EGPA and the MRI findings, the lesion was suspected as the HSP, and the patient was introduced to our department. On admission, the patient could not maintain a standing posture, let alone walking. Muscle weakness in the left hand and both feet and an enhanced patellar tendon reflex were also observed. The patient did not have a bladder bowel disturbance (BBD).

Notable laboratory data are as follows (Table 1): white blood cell (WBC) count of 17300/μL, neutrophils consisted of 92.6%, and the C-reactive protein (CRP) was 3.15. Other differentials included abscess, hematoma, and neoplasms, such as meningioma, were also listed as differential diagnoses.

**Table 1:** Laboratory findings on admission ALTː aspartate aminotransferase; ASTː alanine aminotransferase; BUNː blood urea nitrogen; WBCː white blood cellː RBCː red blood cell

Biochemistry/Immunology	Result	Normal range
Total protein (g/dL)	6.3	6.6-8.1
Serum albumin (g/dL)	3.1	4.1-5.1
AST (U/L)	22	13-30
ALT (U/L)	17	Jul-23
BUN (mg/dL)	28.6	8.0-20.0
Serum creatinine (mg/dL)	0.81	0.46-0.79
C-reactive protein (mg/dL)	3.15	0.00-0.14
Complete bood cell count	Result	Normal range
WBC (×10⁴ /μL)	17.3	3.3-8.6
Neutrophil (%)	92.6	37.0-80.0
Lymphocyte (%)	3.7	11.0-50.0
Monocyte (%)	3.3	4.0-11.0
Eosinophil (%)	0.3	0.0-8.0
RBC (×10⁴ /μL)	510	357-497
Hemoglobin (g/dL)	16	13.7-16.8
Hematocrit (%)	47.1	35.1-44.4
Platelet (×10⁴ /μL)	39.3	13.0-35.0

**Figure 2:**
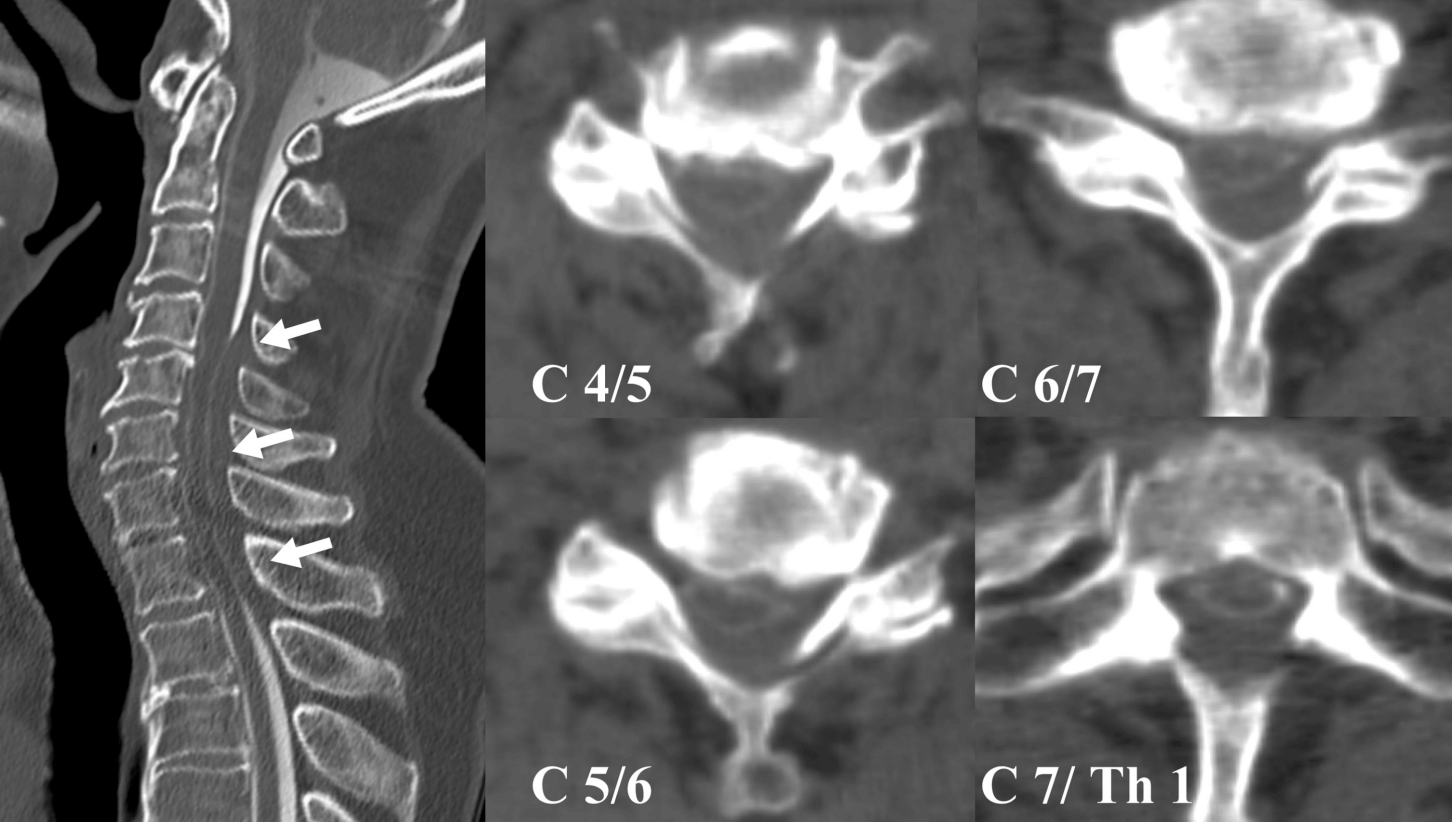
Sagittal and axial images of the myelography CT The defect of contrast enhancement at the C4-Th1 level can be observed, which indicates the cerebrospinal fluid (CSF) blockade (white arrows).

We planned the surgical procedure to decompress the spinal cord and to conduct the biopsy of the dura mater. Since the MRI was poor quality, we planned post-myelography computed tomography (CT) before the surgical intervention. The CT was conducted both as a means of planning for the operation and of scrutinizing the whole spine. The patient could not keep the same posture during the examination because of the severe headache and neck pain, so we managed to continue it under sedation, using an intravenous injection of 2 mg of midazolam. The CT revealed an obstruction of contrast at the cervical dural sac, which indicated a cerebrospinal fluid (CSF) blockade and a compression of the spinal cord (Figure 2).

A few hours after the examination, the blood pressure fell to 68/48 mmHg. Although the patient was conscious, a complete paralysis of both his upper and lower extremities occurred, which seemed to be a tetraplegia. All the deep tendon reflexes and the contraction of an anal sphincter also disappeared.

**Figure 3:**
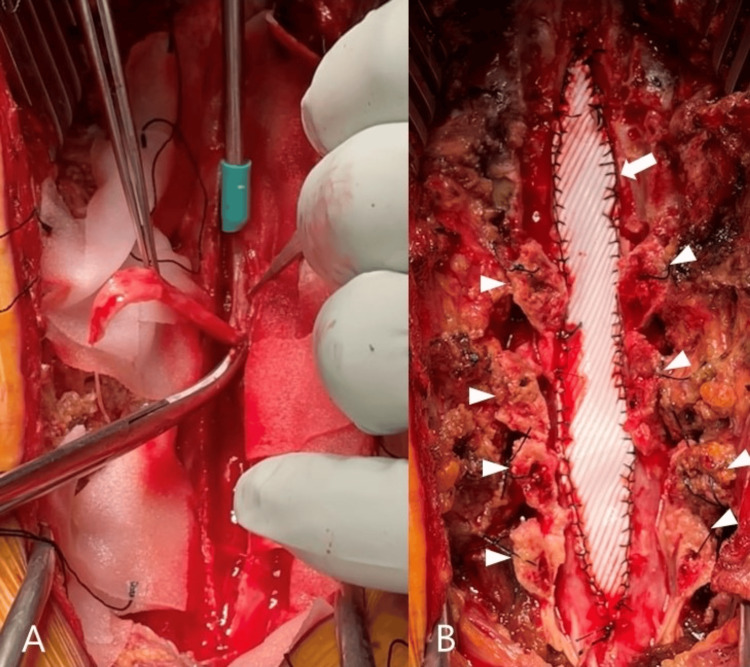
Intraoperative pictures of the cervical spine A: Thickened dura mater is resected; B: Open-door laminoplasty is performed (arrowheads point out the lamina), and the dorsal dura mater is repaired by polytetrafluoroethylene sheet (white arrow).

We diagnosed it as spinal shock due to the cervical spinal cord injury, and an emergency surgery was performed on that day. We performed laminectomy at C3 and Th1-2 and laminoplasty at the C4-7 level. The thickened dura mater at the C4-Th2 level was resected and repaired using an expanded polytetrafluoroethylene sheet (Figure 3).

**Figure 4:**
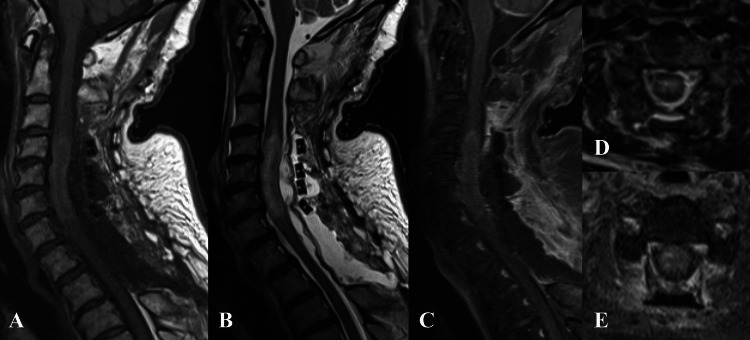
Postoperative gadolinium-enhanced MRI of the cervical spine The dural sac is decently expanded, but the intradural signal change at the C4-Th1 level resides (arrowhead). Aː Sagittal T1 fat-saturated weighted image; Bː Sagittal T2-weighted image; Cː Sagittal gadolinium-enhanced image; D: Axial T2-weighted image at the C4/5 level; E: Axial gadolinium-enhanced image at the C4/5 level

The postoperative MRI revealed that the dural sac was expanded decently, but the broadened intramedullary signal change resided (Figure 4). Specimens resected at the operation showed chronic fibrous tissues and granuloma formation with infiltration of inflammatory cells, which were histopathologically compatible with secondary HP after EGPA.

The tetraplegia (American Spinal Injury Association (ASIA) scale grade A) did not improve postoperatively. The spontaneous respiration did not recover fully, and a tracheotomy was performed postoperatively. Four months after the operation, which was our last follow-up, there was almost no recovery. The tetraplegia persisted, and the patient was still on the ventilator.

## Discussion

Here we presented a case of HP related to ANCA that caused a spinal cord injury after myelography using sedation. Hypertrophic pachymeningitis is an inflammatory disorder that causes the thickening of the cranial or spinal dura mater. Its prevalence is estimated to be 0.949/100,000 people [1]. Hypertrophic pachymeningitis of the spine is relatively rare, with an incidence of 9% compared to the cranial form with 85% [1].

Hypertrophic pachymeningitis is classified as idiopathic or secondary, depending on whether there are any underlying diseases or not. The estimated incidence of idiopathic and secondary HP is about 80% and 20% [2]. Etiologies for secondary HP include autoimmune disorders, infection, and neoplasms. Among autoimmune disorders, ANCA-associated vasculitis and IgG4-related disease are relatively common [3].

However, there are only a few cases that presented EGPA-related HP so far [4-8], and Shiraishi estimates its incidence to be 1.69/10,000,000,000 [8]. Common symptoms of HSP include weakness, sensory disturbance, neck or back pain, ataxia, and BBD. It often shows progressive clinical manifestation, which starts with back pain, radiculopathy, and myelopathy thereafter. Furthermore, about 90% of patients with myelopathy show a progressive clinical course, with their neurological symptoms deteriorating [3]. As for laboratory data, inflammatory markers like erythrocyte sedimentation rate (ESR) and CRP elevate in 75% of cases [1].

Gupta implies that an elevation of these markers suggests a worse clinical prognosis compared to cases otherwise [3]. However, there are not a few cases in which these biomarkers show negative, so these markers lack specificity.

Magnetic resonance imaging is a useful modality for diagnosis. The thickened dura is presented to be hypointense in T1- and T2-weighted imaging and also enhanced with gadolinium contrast. As a differential diagnosis, neoplasms and hematomas should be considered [9, 10].

Histopathological findings are also essential, especially for diagnosing idiopathic HP. It often shows proliferated fibrous collagen tissues with infiltrated lymphoplasmacytic cells, presenting chronic inflammation [11,12].

Specific treatment for the background disease is essential for the secondary HSP: antibiotics for infections and corticosteroids for autoimmune disease. As for idiopathic HSP, corticosteroid is also mainstream for the treatment.

If there is progressive myelopathy, surgical treatment is also an option in order to prevent irreversible neurological damage. Decompression of the spinal cord with laminectomy/laminoplasty is preferred. Resection of the thickened dura as a means of biopsy can be recommended.

Neurological complications like tetraplegia and paraplegia after myelography are quite rare, with only a few case reports documented previously [13,14]. The underlying condition of the spinal canal, such as mass lesions and stenosis resulting from spondylosis or herniation, might increase the risk of the complication.

We hypothesized that the usage of the sedation for myelography had caused the spinal shock since the patient’s neck was mandatorily hyperextended and then the thickened dura mater compressed the spinal cord.

Compression myelopathy at the cervical spine is caused by various factors, among which are told to be static and dynamic factors. Cervical spondylosis comprises a major part of the static factor, while buckling of ligamentum flavum via the neck posture may cause myelopathy as a dynamic factor [15]. In this case, the existence of HP served as a static factor, and the hyperextension of the neck as a dynamic factor, both exacerbated the compression of the spinal cord.

In addition to the compression of the spinal cord, the pressure dynamics of the CSF fluid caused by the injection of contrast media might affect the compression of the spinal cord at the stenotic segment. Even a small amount of contrast injection might worsen the symptoms.

Neurological complications after myelography are often said to be transient, lasting no longer than 48 hours [13]. In this case, however, the neurological deficit became almost irreversible, and the tetraplegia didn’t recover. There are few cases reported of an unexpected spinal cord injury after sedation as well [16,17], which denotes that it is quite a rare condition. We might as well avoid using sedation on patients with severe stenosis at the cervical spine.

## Conclusions

We experienced a case of secondary HP with EGPA, which unexpectedly caused a spinal cord injury due to the hyperextension of the neck. Both the usage of sedation and the myelography itself might affect the neurological deficit. Since the MRI showed poor quality in this case because the patient could not maintain the posture, we conducted post-myelography CT in order to examine the spinal canal more precisely and assess the severity of spinal cord compression. However, the examination eventually caused tetraplegia, a serious complication that is difficult to recover from. Although the necessity of myelography CT is decreasing, there might be some cases in which the examination is considered an option. However, we should avoid conducting myelography and using sedation, especially when the patients suffer severe cervical stenosis.

## References

[1] Yonekawa T, Murai H, Utsuki S (2014). A nationwide survey of hypertrophic pachymeningitis in Japan. J Neurol Neurosurg Psychiatry.

[2] Ashkenazi E, Constantini S, Pappo O, Gomori M, Averbuch-Heller L, Umansky F (1991). Hypertrophic spinal pachymeningitis: report of two cases and review of the literature. Neurosurgery.

[3] Gupta A, Um D, Samant R, Hasbun R, Samudralwar RD, Sriwastava S, Gupta RK (2023). Idiopathic hypertrophic spinal pachymeningitis. J Med Cases.

[4] Izuka S, Yamashita H, Takahashi Y, Kaneko H (2022). Hypertrophic pachymeningitis in eosinophilic granulomatosis with polyangiitis. Mod Rheumatol Case Rep.

[5] Martínez-Piña DA, Calderón-Garcidueñas AL, Gama-Lizárraga E, Enríquez-Peregrino KG, Curiel-Zamudio JM (2024). Hypertrophic pachymeningitis, associated with eosinophilic granulomatosis with polyangiitis, and ANCA-negative serology. Eur J Case Rep Intern Med.

[6] Kiyohara M, Shirai T, Nishiyama S, Sato H, Fujii H, Ishii T, Harigae H (2022). Hypertrophic pachymeningitis development in eosinophilic granulomatosis with polyangiitis at relapse of disease: a case-based review. Tohoku J Exp Med.

[7] Nakano Y, Miyawaki Y, Sada KE (2019). Development of hypertrophic pachymeningitis in a patient with antineutrophil cytoplasmic antibody-negative eosinophilic granulomatosis with polyangiitis. J Clin Rheumatol.

[8] Shiraishi W, Tsujimoto Y, Shiraishi T (2021). Pathological findings of hypertrophic pachymeningitis associated with eosinophilic granulomatosis with polyangiitis. BMJ Case Rep.

[9] Yao A, Jia L, Wang B, Zhang J, Zhang J, Xu B (2019). Idiopathic hypertrophic pachymeningitis mimicking meningioma with occlusion of superior sagittal sinus: case report and review of literature. World Neurosurg.

[10] Park JY, Choi I, Khil EK, Kim WJ, Shin IY (2020). Idiopathic hypertrophic spinal pachymeningitis with spinal cord lesion: a case report. Korean J Neurotrauma.

[11] Ranasinghe MG, Zalatimo O, Rizk E, Specht CS, Reiter GT, Harbaugh RE, Sheehan J (2011). Idiopathic hypertrophic spinal pachymeningitis. J Neurosurg Spine.

[12] Tosa M, Hara M, Morita A (2015). Idiopathic hypertrophic spinal pachymeningitis. Intern Med.

[13] Soliman HM, Arnold PM, Madarang EJ (2013). Post-myelography paraplegia in a woman with thoracic stenosis. J Spinal Cord Med.

[14] Cordier D, Wasner MG, Gluecker T, Gratzl O, Merlo A (2008). Acute paraplegia after myelography: decompensation of a herniatad thoracic disc. Br J Neurosurg.

[15] Development Committee for the Clinical Practice Guidelines on the Management of Cervical Spondylotic Myelopathy (2024). The essence of clinical practice guidelines for cervical spondylotic myelopathy, 2020. Spine Surg Relat Res.

[16] Miller RA, Crosby G, Sundaram P (1987). Exacerbated spinal neurologic deficit during sedation of a patient with cervical spondylosis. Anesthesiology.

[17] Sriganesh K, Ramesh VJ, Veena S, Chandramouli BA (2010). Dexmedetomidine for awake fibreoptic intubation and awake self-positioning in a patient with a critically located cervical lesion for surgical removal of infra-tentorial tumour. Anaesthesia.

